# Breast cancer after thoracic radiotherapy in young patients: what does the radiologist need to know?

**DOI:** 10.1590/0100-3984.2022.0065-en

**Published:** 2023

**Authors:** Bianca Miranda Lago, Stella dos Santos Bello, Guilherme Rocha Melo Gondim, Fabiana Baroni Alves Makdissi, Almir Galvão Vieira Bitencourt

**Affiliations:** 1 A.C.Camargo Cancer Center, São Paulo, SP, Brazil

**Keywords:** Breast neoplasms, Radiotherapy, Mammography, Magnetic resonance imaging, Câncer de mama, Radioterapia, Mamografia, Ressonância magnética

## Abstract

Radiation-induced secondary tumors constitute a rare complication of radiation therapy and typically occur in or near the irradiated area. Women who undergo thoracic radiotherapy before 30 years of age have a significantly greater lifetime risk of developing breast cancer than do those in the general population. It is recommended that a patient who has previously received thoracic radiotherapy with a radiation dose ≥ 10 Gy subsequently undergo annual screening with mammography and magnetic resonance imaging, beginning eight years after the initial treatment or when the patient has reached 25 years of age (whichever comes later). The treatment of secondary breast cancer in this population should be individualized and should be discussed with a multidisciplinary team to avoid toxicity related to the treatment of the primary cancer.

## Abstract (pt)

Os cânceres secundários induzidos por radiação são complicações raras da radioterapia e geralmente ocorrem na área tratada ou próximo a ela. Mulheres com menos de 30 anos de idade tratadas com radioterapia torácica têm risco significativamente aumentado de desenvolver câncer de mama ao longo da vida, em comparação com a população em geral. Para as pacientes tratadas com irradiação torácica com dose igual ou superior a 10 Gy é recomendado rastreamento anual com mamografia e ressonância magnética iniciando oito anos após o tratamento, desde que este início seja após 25 anos de idade. O tratamento do câncer de mama secundário nessa população deve ser individualizado e discutido com equipe multidisciplinar, para evitar toxicidade relacionada ao tratamento do câncer primário.

## INTRODUCTION

Radiation-induced tumors are rare, being observed in less than 1% of patients undergoing radiotherapy. The main risk factor for the development of a radiation-induced secondary tumor is the age at which the primary tumor was treated, younger age at the initial treatment correlating with greater risk. Children are three to six times more susceptible to the carcinogenic effect of ionizing radiation than are adults.

Radiation-induced tumors can be divided into two groups^(1,2)^: those that are sporadic; and those related to hereditary syndromes that increase the risk of cancer. Sporadic radiation-induced tumors are identified on the basis of Cahan’s criteria^(3)^: occurring in the previous radiation field but not present on the images acquired at the beginning of radiotherapy; a mean latency period of 10 years (range, 6 months to 20 years) between exposure to radiation and the development of the secondary tumor; being histologically different from the primary tumor; and occurring in patient with no genetic syndrome that predisposes to cancer. Radiation-induced tumors related to genetic syndromes are mainly related to pathogenic mutation of TP53, which leads to Li-Fraumeni syndrome. The latency period can be shorter for such tumors than for sporadic tumors^(1,2)^.

Thoracic radiotherapy has been successful for the treatment of primary cancers in childhood and adolescence, with a significant increase in relative survival, the ten-year survival rate among such cases having been reported to be approximately 95%^(4)^. However, the radiotherapy increases the risk of secondary cancer, especially breast cancer^(4-10)^. This reaffirms the concept that breast tissue is one of most radiosensitive organs in the human body. Secondary cancer is also the main cause of death among primary cancer survivors^(11)^.

The aim of this study was to review aspects related to the risk of developing secondary breast cancer in young patients undergoing thoracic radiotherapy, as well as current screening recommendations, tumor characteristics, and the peculiarities of cancer treatment in this population.

## RISK OF DEVELOPING BREAST CANCER AFTER THORACIC RADIOTHERAPY

Women undergoing thoracic radiotherapy when they are under 30 years of age are up to eight times more likely to develop breast cancer during their lifetime than are those in the general population^(12)^. Among such women, the risk of developing breast cancer increases five to nine years after the initial treatment, peaking between post-treatment years 15 and 19 and declining thereafter, although the risk remains high until 30-40 years after the initial treatment^(5)^. Horst et al.^(13)^ found that 34% of patients who were ≤ 30 years of age when they were treated for Hodgkin lymphoma (HL) later developed breast cancer, with a median latency period of 21 years (range, 10-30 years) and a median age at breast cancer diagnosis of 43 years (range, 34-66 years). The authors also found that, among the patients who were > 30 years of age when they were treated for HL, only 19% later developed breast cancer, with a median latency period of 18 years (range, 6-29 years) and a median age at breast cancer diagnosis of 53 years (range, 38-79 years).

In a retrospective cohort study designed to identify risk factors for breast cancer in female survivors of primary childhood cancer, including 6,068 women, Kenney et al.^(14)^ demonstrated that the incidence of secondary breast cancer was higher among the women with a family history of breast cancer or thyroid disease and was lower among those exposed to radiation of the pelvis (compared with those exposed to radiation of the thorax). The risk has also been shown to be greater among women who received radiation at younger ages and to increase cumulatively with age^(11,12,15)^. Another factor that has been shown to increase the risk of breast cancer after radiotherapy is the concomitant use of chemotherapy with anthracycline during the treatment of the primary cancer^(16,17)^.

Regarding previous thoracic radiotherapy, the risk of breast cancer has been found to show a linear relationship with the radiation dose, although the risk is lower when there is less breast tissue involved, even when the radiation dose is applied to the entire thorax^(11)^. In addition, fiveand ten-year survival rates remain the same, regardless of the radiation dose or the area irradiated. Moskowitz et al.^(5)^ showed that the risk remains high even if lower radiation doses (10-19 Gy) were used, justifying breast cancer screening in patients receiving such doses, despite the fact that 20 Gy is the cutoff point previously established for breast cancer screening in women having undergone thoracic radiotherapy. It is noteworthy that although the induction of secondary neoplasms is a stochastic event (i.e., the risk increases in proportion with the applied dose and the volume of irradiated tissue), low doses and small volumes do not completely eliminate the risk. Thoracic radiotherapy for lymphoma, for example, currently uses lower doses and volumes than previously standardized, which could make the risk of radiation-induced tumors lower than that observed in historical series.

## SCREENING RECOMMENDATIONS

Because women who have undergone thoracic radiotherapy in childhood and adolescence are at an increased risk of developing breast cancer before entering the age range usually indicated for screening, the recommendations for such women should be different from those applied in the general population. Mammography, the most widely used method of screening for breast cancer, is less sensitive in young women because breast density is greater in that population. As detailed in Table 1, various authors have demonstrated the benefit of incorporating magnetic resonance imaging (MRI) in the screening strategy for young women who have previously undergone thoracic radiotherapy, showing that the combination of mammography and MRI is more sensitive than is mammography alone^(18-22)^. The American College of Radiology^(23)^. and the Brazilian College of Radiology and Diagnostic Imaging, together with the Brazilian Breast Disease Society and the Brazilian Federation of Gynecology and Obstetrics Associations^(24)^, recommend that such women be screened annually with mammography and MRI.

**Table 1:** Sensitivity of mammography, MRI, and the combination of the two for diagnosing secondary breast cancer during screening in women who had undergone thoracic radiotherapy before 30 years of age.

Study	N	Sensitivity		
		Mammography	MRI	Mammography + MRI
Sung et al.**^(18)^**	91	60%	70%	100%
Ng et al.**^(19)^**	148	68%	67%	94%
Freitas et al.**^(20)^**	98	69%	92%	100%
Tieu et al.**^(21)^**	96	70%	80%	100%
Ehrhardt et al.**^(22)^**	1,467	54%	69%	86%

In 2019, the International Late Effects of Childhood Cancer Guideline Harmonization Group published updated recommendations for breast cancer screening in women treated for cancer in childhood, adolescence, or young adulthood^(25)^. The recommendations include annual screening for breast cancer, starting at age 25 or eight years after radiotherapy (whichever comes later), for patients undergoing thoracic radiotherapy at a dose ≥ 10 Gy before 30 years of age and the continuation of such screening until at least 60 years of age. An additional recommendation is that screening for breast cancer should also be considered in patients who underwent radiotherapy only of the upper abdomen. Those practices would allow early detection of the disease, improving the prognosis, given the fact that the therapeutic options are often limited, because such women have already been submitted to chemotherapy and radiotherapy during the treatment of the primary tumor^(21)^.

Because of the difficulty in gathering a considerable number of women who fit the profile for studies designed to validate these screening indications, some authors have developed mathematical models to assess the benefit of early screening in HL survivors from the age of 25 onward^(26,27)^. Those models demonstrated that early screening with mammography and MRI would reduce the absolute risk of death from breast cancer in this population by as much as half. The additional benefit of mammography over MRI was very small in those studies. However, the models used did not consider ductal carcinoma *in situ* (DCIS) as part of the natural history of breast cancer, assuming that it would not contribute to mortality. That omission could have interfered with the results of mammographic screening. In both studies, a higher number of false-positive screening results were observed when MRI was included, although the values were considered to be acceptable.

## CHARACTERISTICS OF RADIATION-INDUCED BREAST CANCER

The most common malignant breast neoplasms after chest radiotherapy are invasive carcinoma not otherwise specified (formerly known as invasive ductal carcinoma) and DCIS. In this population, most tumors are diagnosed at an earlier stage^(28)^ and express hormone receptors. In one study of secondary breast tumors in women who had received radiotherapy in childhood or young adulthood, Demoor-Goldschmidt et al.^(29)^ found that 70% of the secondary tumors had estrogen receptors and 64% had progesterone receptors. However, compared with cases of breast cancer in the general population, cases of such radiation-induced secondary tumors have been shown to have a worse prognosis, with higher rates of bilaterality and a higher grade, as well as being less likely to express hormone receptors and more likely to present the triple-negative subtype^(11,30)^. Horst et al.^(30)^ found that 39% of patients with breast cancer who had undergone thoracic radiotherapy presented the triple-negative subtype, compared with only 14% of the patients with sporadic breast cancer.

**Figure 1:**
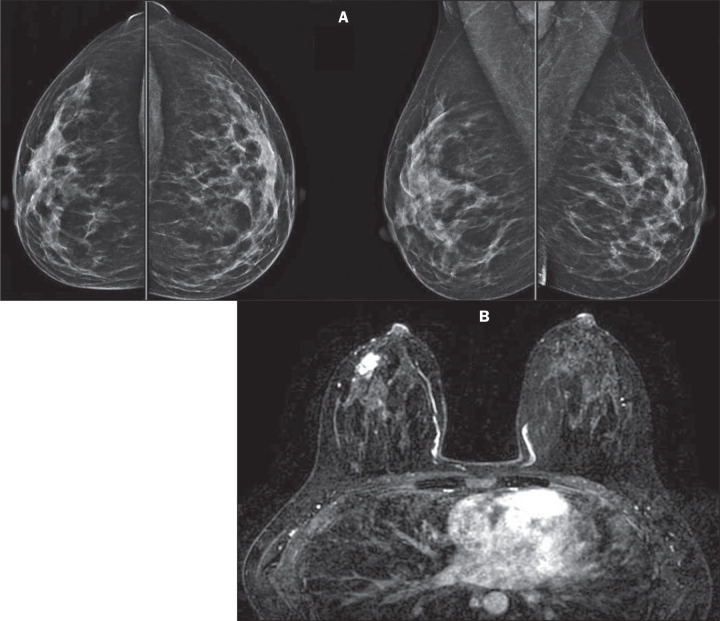
A 35-year-old patient who had previously undergone thoracic radiotherapy and subsequently underwent regular screening with mammography and MRI. A: Mammography showing heterogeneously dense breasts, with benign-looking calcifications, without evidence of masses, classified according to the Breast Imaging Reporting and Data System (BI-RADS) as a category 2 (benign) finding. B: Breast MRI showing a mass with irregular margins and rapid early enhancement in the right breast, measuring 2.5 cm, classified as a BI-RADS category 4 (suspicious) finding. A biopsy confirmed a diagnosis of invasive ductal carcinoma, categorized as the luminal B subtype, with pT2pN1a surgical staging.

**Figure 2:**
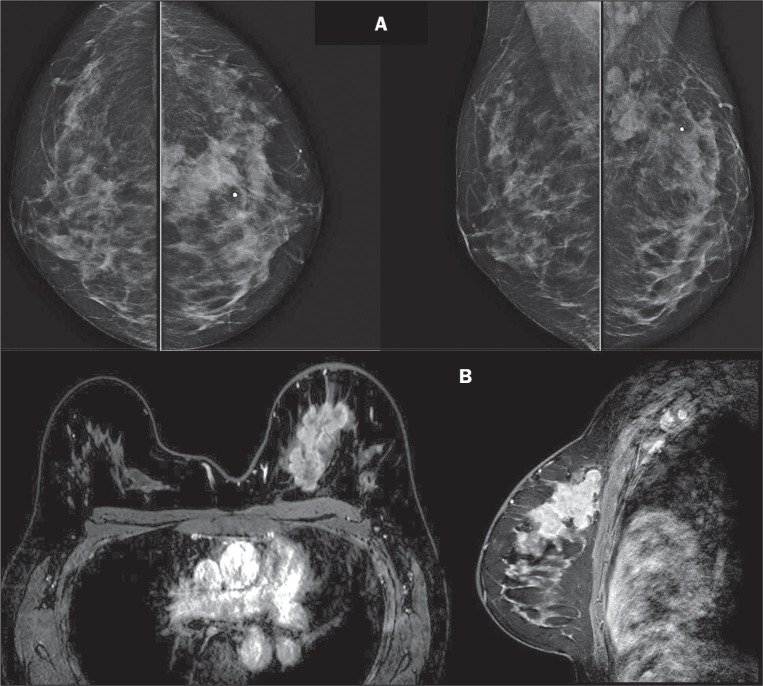
A 30-year-old patient who had undergone mantle-field (thoracic) radiotherapy for the treatment of HL at 11 years of age and did not undergo subsequent screening, presenting with a palpable mass in the left breast. A: Diagnostic mammography showing an irregular mass in the posterior third of the junction of the upper quadrants of the left breast, measuring 6.0 cm, accompanied by architectural distortion and calcifications, together with atypical ipsilateral axillary lymph nodes, classified according to the BIRADS as a category 5 (probably malignant) finding. B: Breast MRI confirming the irregular mass occupying the upper inner quadrant of the left breast, measuring 7.8 cm. Biopsy confirmed a diagnosis of invasive ductal carcinoma, categorized as the luminal B subtype with overexpression of human epidermal growth factor receptor 2. A positron-emission tomography/computed tomography scan, performed for staging, showed lung metastases (clinical staging, T3N2M1).

The imaging findings at diagnosis of radiation-induced breast cancer are similar to those of breast cancer in the general population, depending on the timing of the diagnosis. In a multicenter study conducted at hospitals in the United States and Canada, Elkin et al.^(31)^ demonstrated that most radiation-induced tumors were diagnosed during screening. In another multicenter study, carried out in France, Demoor-Goldschmidt et al.^(32)^ found that most such tumors were diagnosed in the symptomatic phase. In general, patients diagnosed during the screening process tend to have smaller, earlier stage tumors (Figure 1), whereas those diagnosed in the symptomatic phase tend to have tumors that are larger and more aggressive (Figure 2).

Most radiation-induced tumors present as masses, which are generally better characterized on MRI, although some can be identified only by mammography, so the two methods should be used in conjunction^(18-22)^. As shown in Table 1, the sensitivity for the diagnosis of breast cancer is higher when the screening is performed with mammography and MRI than when it is performed with mammography or MRI alone. In the study carried out by Horst et al.^(13)^, approximately 30% of malignant neoplasms were DCIS, identified by the presence of calcifications on mammography, although not all of the patients in that study underwent MRI. In a study conducted by Sung et al.^(18)^, who also employed mammography, MRI, and the combination of the two, 30% of radiation-induced malignant tumors presented only as a mass, 30% only as calcifications, 20% as a mass together with calcifications, and 20% as non-mass enhancement.

## PECULIARITIES OF THE TREATMENT OF RADIATION-INDUCED BREAST CANCER

The surgical treatment most often used in cases of radiation-induced tumors is mastectomy, mainly because the risk of new tumors in the previously irradiated breast tissue persists over the years and because there can be intolerance to new radiotherapy, although radiotherapy can be performed safely in selected cases^(33)^. Intraoperative radiotherapy and partial breast irradiation can also be performed for early-stage secondary breast cancer after breast-conserving surgery. It is known, however, that re-irradiation has possible side effects, including radionecrosis; therefore, breast reconstruction with healthy, well-vascularized autologous tissue is recommended, and silicone prostheses can be an option in patients with mild radiation-induced alterations^(13)^.

The systemic treatment of secondary radiation-induced breast cancer is similar to that of primary breast cancer. Given the risk of cardiotoxicity, attention should be paid to the cumulative dose of anthracycline in the first and second cycles of treatment. However, depending on the biological characteristics of the breast cancer, adjuvant chemotherapy, including anthracycline therapy, can also be administered, even in patients previously exposed to that class of medication, with good tolerability and no acute cardiotoxicity^(34)^. In such cases, there must be a multidisciplinary approach for women who survive the primary tumor and develop a secondary breast tumor after thoracic radiotherapy, who should be offered the same options for effective treatment that are offered to women with sporadic breast cancer.

## CONCLUSIONS

Radiation-induced secondary cancers are rare complications of radiotherapy and usually occur in or near the irradiated area. Women receiving thoracic radiotherapy in childhood, adolescence, or young adulthood (especially for the treatment of HL) are at increased risk of developing breast cancer during their lifetime. Such women should undergo annual screening with mammography and MRI starting eight years after treatment, assuming that they are at least 25 years of age at that time. Screening allows early detection of the disease, improving the prognosis, given that previous radiotherapy limits the therapeutic options.
